# CD138^-^ multiple myeloma cells express high level of CHK1 which correlated to overall survival in MM patient

**DOI:** 10.18632/aging.104066

**Published:** 2020-11-10

**Authors:** Dong Wu, Peihua Zhang, Fangmei Li, Ying Shen, Hongli Chen, Yuandong Feng, Aili He, Fangxia Wang

**Affiliations:** 1 Department of Hematology, The Second Affiliated Hospital of Xi’an Jiaotong University, Xi’an, Shaanxi, China

**Keywords:** multiple myeloma, CD138, CHK1, cancer stem cell, bioinformatics

## Abstract

Multiple myeloma (MM) is a disease in which abnormal plasma cells proliferate and secrete monoclonal immunoglobulin in the bone marrow. The main characteristic of plasma cells is the expression of the cell surface antigen syndecan-1 (CD138). However, the expression of CD138 is limited to terminally differentiated plasma cells during B cell development. A small subpopulation (2~5%) of human MM cells that lack CD138 expression has been shown to possess enormous proliferation potential *in vitro* experiment and in animal models, and they also can differentiate into CD138^+^ plasma cells. Thus, this small subset of MM cells was regarded as myeloma cancer stem cell (MCSC). However, its characteristics associated with the pathogenesis of MM remain unclear. In this study, we analyzed the gene expression data of CD138 cell lines downloaded from Gene Expression Omnibus (GEO) database. Limma package in RStudio was used to identify differentially expressed genes (DEGs). Genes enrichment and protein-protein interaction (PPI) network analysis were performed on DAVID and STRING databases. Furthermore, overall survival (OS) analysis in MM patient was utilized to screen out the hub-genes closely associate with the MM pathogenesis process. Hub-genes expression validation and receiver operating characteristic curve (ROC) analysis was performed in different stages of plasma cell disorder diseases. Finally, we verified these findings in MM patient samples. Through integrated bioinformatics analysis of MM CD138^-^ and CD138^+^ cell lines, we found that CDC7, CDK1, and CHK1 are highly expressed in CD138^-^ MM cells. These genes are crucial in the G2/M phase of the cell cycle pathway, which is closely related to the malignant proliferation in various tumor cells. Of note, we found that patients with high expression of CDC7, CDK1, and CHK1 had shorter overall survival time. The expression of CHK1 was significantly increased in MM cells compared with normal plasma cell (NPC) and MGUS. More importantly, we further clarified that the expression of CHK1 in release/refraction MM (R/R MM) has obviously increased compared with new diagnosed MM (ND MM).

## INTRODUCTION

Multiple myeloma (MM) is an incurable malignant plasma cell disease and is the second most common hematology malignant tumor [1]. Numerous advances are available for the treatment of MM, such as immunomodulators drugs (IMiDs), proteasome inhibitors (PI), monoclonal antibodies, autologous stem-cell transplantation (ASCT), and chimeric antigen receptor T(CAR-T) cell therapy, [2–4]. The utilization of these therapies has dramatically improved the overall survival of MM patients. However, almost all of the patients ultimately relapse and drug resistance [5]. To understand this issue, a great many works have been devoted to explore the oncogenesis of MM [6].

MM cells have obvious genetic heterogeneity. Only a small number of myeloma cells have the ability of pathogenicity and tumorigenic. These cells are called multiple myeloma "stem cells" [7]. It is currently believed that these “stem cells” should responsible for the initiation, maintenance, and relapse of MM. The character of plasma cells is the expression of syndecan-1 (CD138), but the expression is highly specific both in MM cells and plasma cells of patient specimens. Moreover, during normal B cell development, CD138 is only expressed on end-stage differentiated normal plasma cells, but not in highly proliferating plasma cells and all early stages B cell [8, 9]. Previous studies have detected that, when compared to CD138^-^ MM plasma cells, the proliferation of CD138^+^ plasma cells are inefficient. Furthermore, CD138^-^ MM plasma cells have the ability of carcinogenic and drug resistance, meanwhile these CD138^-^ plasma cells can differentiate into CD138^+^ plasma cells [7, 10]. However, some scholars have different opinions, H. Svachova et al. showed that CD138^+^ MM plasma cells express Nestin protein, which is the hallmark of cancer stem cell. Subsequently, they proved that the expression of Nestin in CD138^+^ cells throughout multistep pathogenesis of MM [11, 12]. Therefore, a better understanding of the distinction between CD138^-^ and CD138^+^ MM plasma cellular characterization will help to develop new targets and strategies for the prognosis and treatment of MM.

In this study, though integrated bioinformatics we found the differential expression of genes in CD138^-^ and CD138^+^ MM cell lines. Furthermore, we validated our findings in MM patient samples which provided new therapeutic targets to MM.

## RESULTS

### Screening of DEGs in CD138^-^ and CD138^+^ cell lines

**Figure 1:**
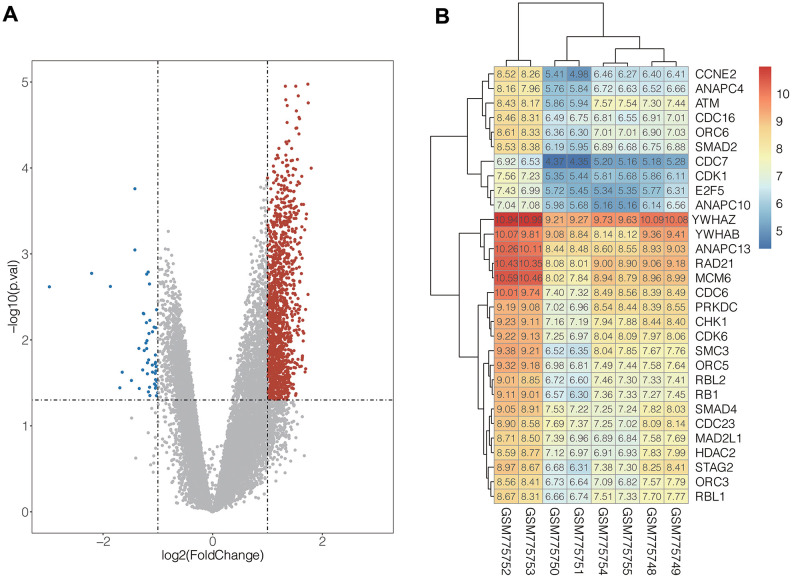
(**A**) Volcanic map of DEGs distribution. The abscissa of volcano map is the logarithmic value of the fold change (FC) of each sample, and the ordinate is the logarithm of 10 corresponding to the P value of the corresponding sample Negative values, the red and blue dots respectively represent genes that are up-regulated and down-regulated, and the grey dots represent genes that are not significantly different. (**B**) The expression heat map of the genes in cell cycle pathway. Red represents high gene expression and blue represents low gene expression.

In the present study, integrated bioinformatics analysis was performed on the GSE31305 dataset, which contains CD138^-^ and CD138^+^ human MM cell lines. After systematic data standardization, 18892 DEGs were identified which included 8608 up-regulated genes and 10284 down-regulated genes. Among these DEGs, 1318 up-regulated and 48 down-regulated genes were significant (| log_2_ FC |> 1 and p <0.05) (Figure 1A).

### Functional analysis of differential genes

**Figure 2:**
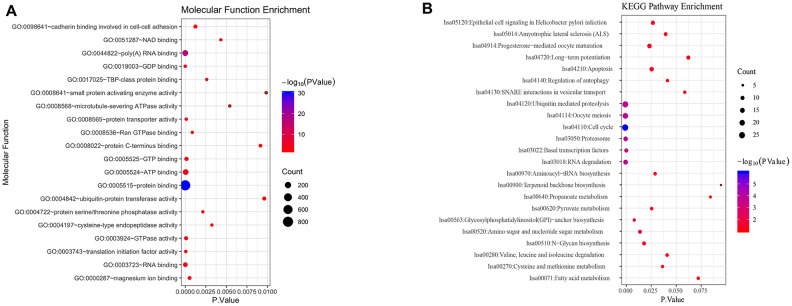
**Functional analysis of differential genes.** (**A**) Molecular function analysis of differential genes. (**B**) KEGG signal pathway of differential genes.

To understand biological function roles of the above identified DEGs, the online biological tools DAVID database and KEGG database were used for enrichment analysis. Top enriched GO terms and KEGG pathways were listed in Table 1 and Table 2 (Supplementary Table 2). In molecular function analysis (Figure 2A), differential genes are mainly concentrated in protein binding. In KEGG analysis (Figure 2B), it was found that differential genes are mainly involved in cell cycle pathways. The volcano map of all differentially expressed genes in cell cycle pathway is shown in Figure 1B (Supplementary Table 1).

**Table 1:** List of top enriched gene ontology terms of up-regulated and down-regulated DEGs.

**Direction**	**Category**	**Term**	**Description**	**Count**	**PValue**
Upregulated	BP	GO:0007062	sister chromatid cohesion	35	9.29E-15
Upregulated	BP	GO:0007067	mitotic nuclear division	53	1.05E-12
Upregulated	BP	GO:0051301	cell division	65	1.86E-12
Upregulated	BP	GO:0015031	protein transport	69	6.70E-12
Upregulated	BP	GO:0051436	negative regulation of ubiquitin-protein ligase activity involved in mitotic cell cycle	22	1.13E-08
Upregulated	MF	GO:0005515	protein binding	808	6.68E-31
Upregulated	MF	GO:0044822	poly(A) RNA binding	158	1.85E-17
Upregulated	MF	GO:0019003	GDP binding	16	3.02E-06
Upregulated	MF	GO:0003723	RNA binding	67	1.16E-05
Upregulated	MF	GO:0005524	ATP binding	145	5.32E-05
Upregulated	CC	GO:0005829	cytosol	407	6.62E-36
Upregulated	CC	GO:0005654	nucleoplasm	326	4.82E-24
Upregulated	CC	GO:0005739	mitochondrion	159	6.60E-12
Upregulated	CC	GO:0005783	endoplasmic reticulum	112	9.03E-12
Upregulated	CC	GO:0016020	membrane	231	3.10E-11
Downregulated	BP	GO:0050911	detection of chemical stimulus involved in sensory perception of smell	9	2.29E-07
Downregulated	MF	GO:0004984	olfactory receptor activity	9	1.25E-07
Downregulated	MF	GO:0004930	G-protein coupled receptor activity	9	5.39E-06
Downregulated	CC	GO:0045095	keratin filament	6	6.82E-07

**Table 2:** List of top enriched KEGG terms of up-regulated and down-regulated DEGs.

**Direction**	**Category**	**Term**	**Description**	**Count**	**PValue**
Upregulated	KEGG	hsa04110	cell cycle	30	1.01E-06
Upregulated	KEGG	hsa04141	Protein processing in endoplasmic reticulum	34	1.15E-04
Upregulated	KEGG	hsa03013	RNA transport	30	1.38E-04
Upregulated	KEGG	hsa03050	proteasome	13	1.57E-04
Upregulated	KEGG	hsa04114	oocyte meiosis	22	2.07E-04
Downregulated	KEGG	hsa04740	olfactory transduction	8	1.29E-06

### PPI network analysis and hub-gene selection

**Figure 3:**
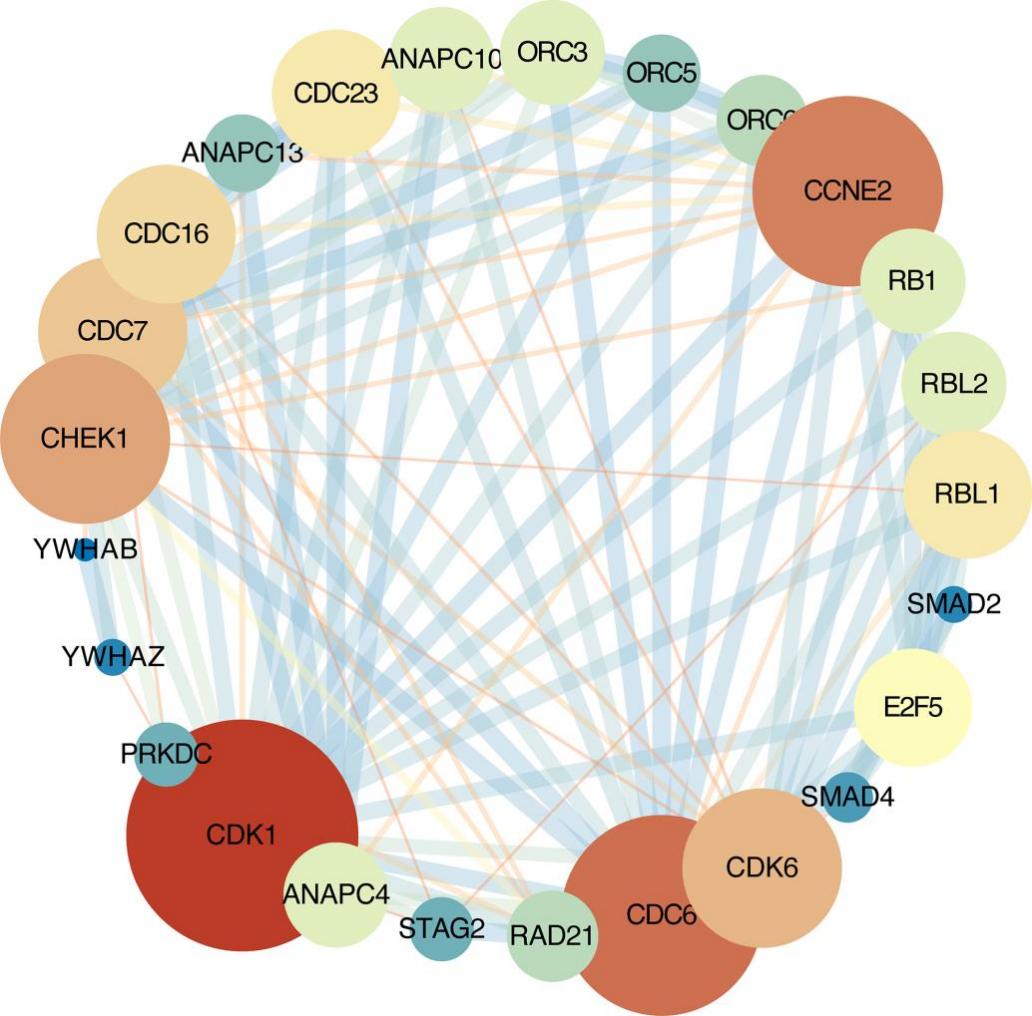
**Protein-protein interaction network.** Each node represents a gene; Node size represents the degree value; Edge size represents the combined score; Low value to blue and high value to red.

The STRING online database was used analyze the DEGs contained in the cell cycle pathway. This pathway contains 30 nodes and 172 edges, average node degree:11.5. Network nodes represent genes and edges represent protein-protein associations. Cytoscape software were used to visualization [13] (Figure 3).

### Overall survival analysis

**Figure 4:**
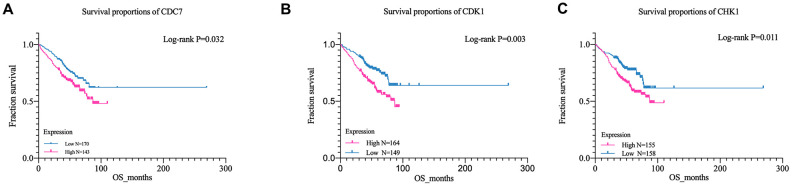
**Kaplan-Meier survival curves of MM patient.** (**A**) Kaplan-Meier analysis for overall survival of CDK1; (**B**) Kaplan-Meier analysis for overall survival of CDC7;(**C**) Kaplan-Meier analysis for overall survival of CHK1.

GSE24080 was selected for overall survival analysis by using survival package in Rstudio, 313 MM patient samples with clinical and overall survival information were divided into high expression group and low expression group according to the average expression of each gene. The result suggested that the expression of cell division cycle 7 (CDC7), cyclin-dependent kinase 1 (CDK1), and checkpoint kinase 1 (CHK1) genes among cell cycle pathway were closely related with the overall survival time of patients, and the OS of the low-expression group was significantly prolonged than that of the high-expression group (CDC7, P = 0.032, CDK1, P = 0.003; CHK1, P = 0.011) (Figure 4A–4C). However, due to the lack of risk stratification of MM patients in the GSE24080 dataset, the relationship between these three genes and MM risk stratification cannot be calculated.

### Expression of hub-genes in different plasma cell diseases

**Figure 5:**
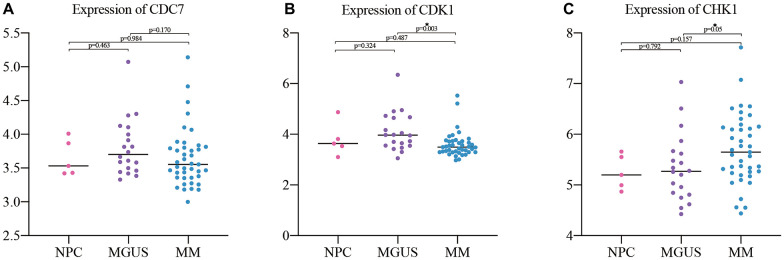
**Expression validation of the hub genes in NPC (5), MGUS (20) and MM (41).** (**A**) expression of CDC7; (**B**) expression of CDK1; (**C**) expression of CHK1.

The GSE47552 dataset was selected to verify the expression of CDC7, CDK1, and CHK1 at different stages of plasma cell diseases. The results showed that there was no significant difference between them when compared MM with NPC and MGUS, but the expression of CHK1 has statistically significant difference compared MM with NPC and MGUS (Figure 5A–5C).

### Receiver operating characteristic curve analysis

**Figure 6:**
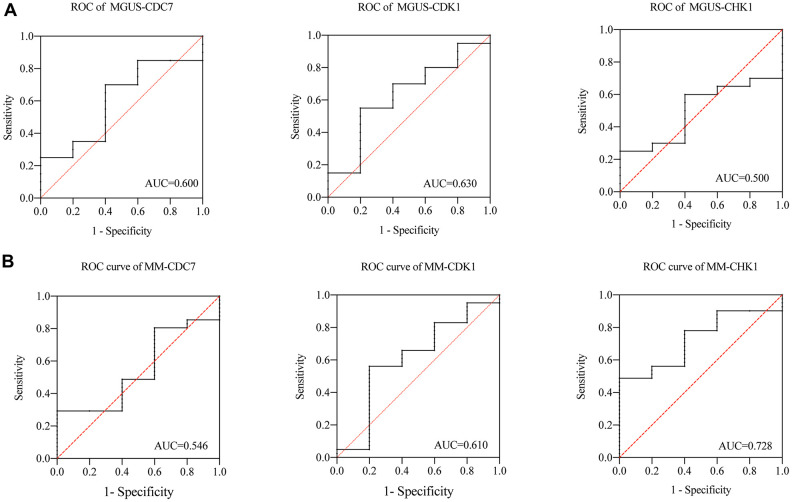
****(**A**) ROC of CDC7, CDK1 and CHK1 in MGUS, (**B**) ROC of CDC7, CDK1 and CHK1 in MM.

we subsequently analyzed the ROC of MGUS and MM. As shown in the Figure 6, in the MGUS, the area under the curve (AUC) of CDC7, CDK1, and CHK1 are 0.600, 0.630, and 0.500 (Figure 6A). In MM, the AUC of CDC7, CDK1, and CHK1 are 0.546, 0.610, and 0.728 (Figure 6B). That means the diagnostic value of CDC7 and CDK1 in MGUS and MM is not significantly different, while CHK1 has an apparent difference between MGUS and MM.

### The expression of CHK1 in MM patients

**Figure 7:**
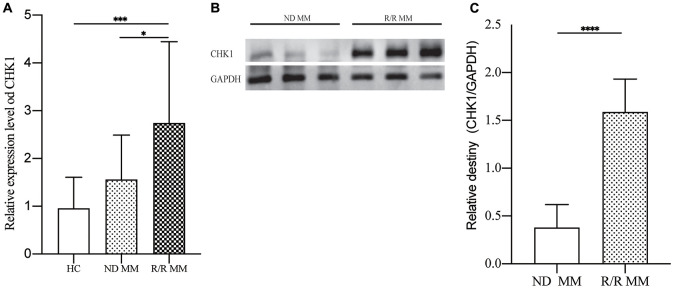
**Verification of CHK1 expression in HC, ND MM, and R/R MM samples.** (**A**) mRNA expression of CHK1 in HC, NDMM and R/R MM samples were analyzed using qRT-PCR. The mRNA expression level of CHK1 was showed by relative expression level. (**B**) Western blot analysis of the protein expression of CHK1 in ND MM, and R/R MM samples. (**C**) Results of western blot analysis showing a drastic increase in R/R MM. P< 0.05 was considered statistically significant (*p<0.05, **P<0.01, ***P<0.001, ****P<0.0001).

To verify our findings, we tested the MM patients mRNA expression of CHK1 and found that there was no statistical difference between HC and ND MM, but there are significant statistical differences when compared R/R MM with HC (*P*=0.0009) and ND MM (*P*=0.0267, Figure 7A). Then we measured the protein expression of CHK1 between ND MM and R/R MM. The results showed that the expression of CHK1 in the R/R MM was obviously increased than that of ND MM (*P*<0.0001, Figure 7B, 7C).

## DISCUSSION

Multiple myeloma (MM) is characterized by proliferation of plasma cells, which secret a great many of monoclonal immunoglobulins [14]. The expression of cell surface antigen CD138 is the feature of plasma cells. However, CD138 is expressed in the terminal differentiation stage of plasma cells, but lost in the early stages of B cell and highly clonal plasma cells [15, 16]. Furthermore, recently study by Akhmetzyanova et al. shows that CD138^+^ cells are mainly related to the proliferation and enhance the sensitivity of IL-6 receptor, and CD138- myeloma cells are mainly involved in the migration process. The expression of CD138 in myeloma can be shed and recycled according to the different serum nutritional environment, so as to facilitate the adaptation of myeloma cells to the environment during the initiation and migration process [17]. Thus, it is necessary to further study the difference between CD138^+^ and CD138^-^ MM cells. CD138^-^ cells account for a small proportion of MM cells, and their characteristics of tumor stem cells in MM have been reported in previous study. Reghunathan et al found that CD138^-^ cells have many common characteristics with normal hematology stem cells, including self-renewal, increase the activation of aldehyde dehydrogenase 1 (ALDH1) and differentiated into CD138^+^ plasma cells [18]. Such a small number cells owning the characteristics of tumor stem cells were also found in many other hematology malignity diseases, such as chronic myelogenous leukemia (CML), acute myeloid leukemia (AML), and acute lymphocytic leukemia (ALL) [19–22]. Therefore, studying the differences in gene expression and biological functions of CD138^-^ and CD138^+^ MM cell lines can help us further understand the progress of MM.

In order to identify the characteristics of CD138^-^ MM malignant profiling cells, integrated bioinformatics analysis has been performed with the GEO datasets, GSE24080 dataset, containing CD138^+^ and CD138^-^ cell lines. First of all, based on the gene expression profiles of the above datasets, 3756 significantly DEGs were identified, then GO and KEGG enrichment analysis found that these DEGs were mainly concentrated in the cell cycle related pathways. Subsequently, we analyzed biological functional and PPI of the DEGs in the cell cycle. Through overall survival analysis, three hub-genes in cell cycle pathways were filtered out, including CDC7, CDK1, and CHK1. The analysis of OS of above hub-genes in MM patients showed that patients with high expression of CDC7, CDK1 and CHK1 had a poor overall survival time.

Because of MGUS can progress to MM, and there are overlaps in cancer related genes between MGUS and MM [23]. In order to further explore the relationship among different states of plasma cells disorder, the expression of CDC7, CDK1 and CHK1 was verified in the gene expression dataset of NPC, MGUS, and MM. The results showed that the expression of CHK1 in MM patients increased compared with NPC and MGUS but p = 0.084, which may be due to insufficient sample size in GSE47552. We will further expand the sample size for verification in the following experiments. Subsequently, the ROC analysis of CDC7, CDK1, and CHK1 in MGUS and MM were conducted. The result suggested that CHK1 could act as a reliable prognosis indicator in MM. In order to verify these findings from datasets, we validated our results in MM patient samples, and the results showed that the expression of CHK1 in mRNA and protein was obviously increased in R/R MM than ND MM.

**Figure 8:**
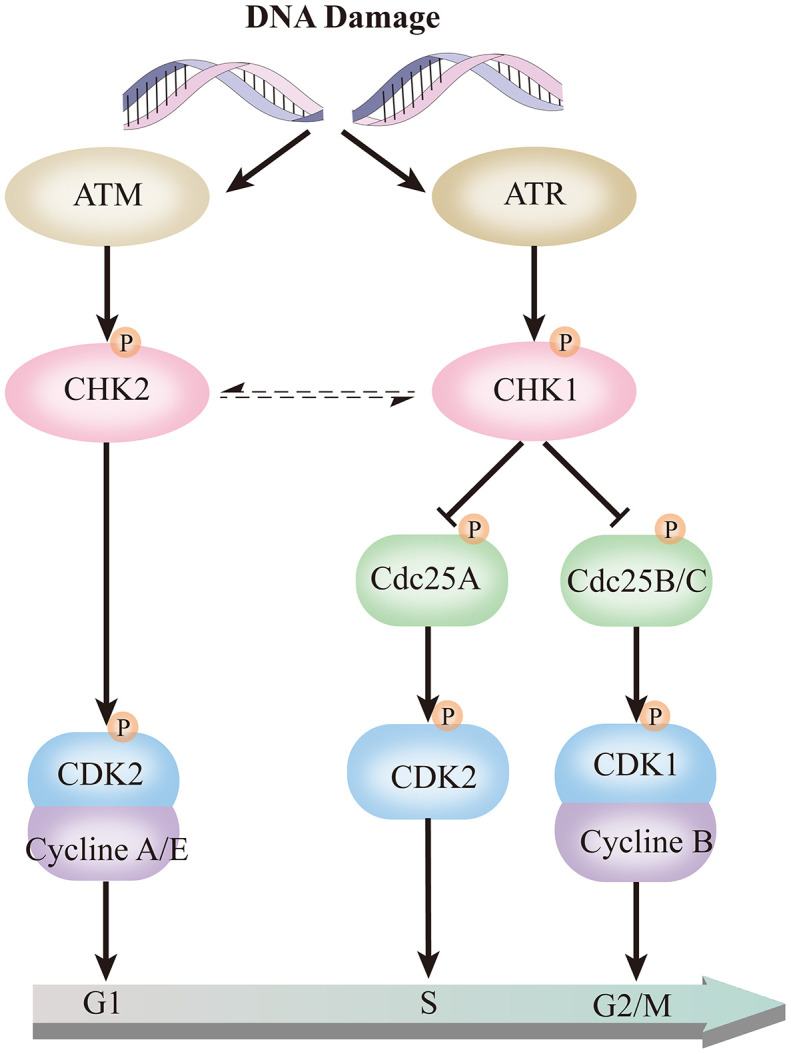
**ATR-CHK1 and ATM-CHK2 in the cell cycle pathway.**

Through the above analysis, it was found that the high expression of CHK1 in CD138^-^ cells may play an essential role in the pathogenesis and maintenance of MM. CHK1 protein kinase regulates the G2/M phase transition in the cell cycle pathway, which is significant in gene replication and transcription. The transmission of genetic information is critical for cell survival, not only does it require the accurate transmission of genetic information, but it also needs to respond and repair in time when DNA is damaged [24]. Genomic instability is a common feature of cancer cells and contributes to the accumulation of oncogenic mutations [25]. DNA damage response (DDR) is a crucial factor in the development and treatment of various cancers. DNA damage causes cell cycle delay, mainly in G1/S and G2/M transitions, and causes a decrease in the rate of DNA synthesis [25]. Cell responses to DNA damage are mainly coordinated by two different protein kinase signal transducers Ataxia Telangiectasia Mutated (ATM) and Ataxia Telangiectasia and Rad3-Related kinase (ATR). ATM-CHK2 pathway primarily responds to DNA double-strand breaks (DSBs), whereas the ATR-Chk1 pathway recognizes extensive DNA abnormalities such as single-stranded DNA (ssDNA), DSBs end resection, DNA replication inhibition, and inter-strand DNA crosslinking [26]. In ATR-CHK1 signaling pathway, ATR activates CHK1 by phosphorylation, and the activation of CHK1 further phosphorylates downstream Cdc25A, limits its ability to drive progression during the S phase. CHK1 also can causes Cdc25B/C phosphorylation, to degrade or release the nucleus, thereby preventing the activation of CDK1 and CDK2, inactivating CDK2 and CDK1 causes G1/S and G2/M phase cell cycle arrest (Figure 8) [26–28]. Cells undergo DNA repair and maintain genome integrity and stable promote cell survival. Similar to the ATR, ATM controls the phosphorylation of p53, BRCA1, and CHK2, which play a crucial role in the ATM-CHK2 pathway [29–31]. CHK1 and CDK1 are hub genes in the ATR-CHK1 pathway. Consistent with our findings, the expression of these two genes in both CD138 cell lines and patient specimens are statistically significant, which further confirmed that this pathway was closely related to the initiation and maintenance of MM.

CHK1 protein kinase can mediate cellular DNA damage response and repair. On the one hand, for normal cells, it does reduce the risk of malignant cell proliferation caused by DNA mutations. On the other hand, for tumor cells, CHK1 protein kinase also can repair DNA damage caused by various anticancer therapies [32]. High expression of CHK1 has been detected in numerous human tumors. Including breast cancer, colorectal cancer, cervical cancer, etc. [33–35]. It was also found that the higher expression of CHK1 is closely related to the relapse and drug resistance in tumors [36] and targeting CHK1 with specific inhibitor has been shown pro-apoptotic effect on CD138+ myeloma cells, but not unapparent in CD138- myeloma cells [37]. In short, the high expression of CHK1 makes tumor cells highly resistant to DNA damage induced by chemotherapy drugs and the severe tumor microenvironment. Eventually, these tumor cells dominate the body, creating clonal tumors that are more malignant and resistant.

Collectively, we identified a dramatical distinction gene expression between CD138^-^ and CD138^+^ plasma cells. These DEGs are mainly concentrated in the ATR-CHK1 cell cycle pathway, which is closely related to the clonal proliferation characteristics of CD138^-^ MM plasma cells. Multiple myeloma cells expressed high levels of CHK1, which correlated to overall survival time in MM patient. These findings provide clues for subsequent research, and also provide new targets for MM prognosis and treatment.

## MATERIALS AND METHODS

### Patients and samples

Bone marrow specimens were obtained from newly diagnosed MM (ND MM) and refractory/relapse MM (R/R MM) patients at the department of hematology, Second Affiliated Hospital of Xi’an Jiaotong University, from 2019 to 2020. This study was approved by the Medical Ethics Committee of the Second Affiliated Hospital of Xi’an Jiaotong University and written informed consent was obtained from all parents or guardians. The diagnosis, stage and risk status of MM were made in accordance with the National Comprehensive Cancer Network (NCCN) (2020 version 4) and mSMART 3.0. Heathy donor bone marrow mononuclear cells (BMNCs) samples were used as health control (HC). All samples were isolated using lymphocyte separation liquid to harvest total cellular RNA, then stored at -80°C.

### Selection of GEO datasets and data processing

The gene expression profiles of CD138 cell lines were downloaded from the GEO database (http://www.ncbi.nlm.nih.gov/geo/). The microarray data with accession number GSE31305 was based on the GPL570 platform (Affymetrix Human Genome U133 Plus2.0 Array, Affymetrix, Santa Clara, CA, USA). GSE31305 dataset contains two kinds of human MM cell lines: RPMI-8226 and NCI-H929, both of them included CD138^-^ and CD138^+^ MM cell lines and have two replicates. When multiple expression levels corresponded to one specific gene, take an average of all expression-levels for that gene.

### Identification of DEGs

To investigate DEGs among CD138^-^ and CD138^+^ cell lines, the Limma package [38] in RStudio (Version 1.2.1335) was used to identify differential expressed genes, |log_2_ FC (fold change) |> 1 and *P* <0.05 were used as the cut-off criteria.

### GO and KEGG pathway enrichment analysis

To identify the significant biological roles of these DEGs, Gene ontology (GO) enrichment analysis was performed using DAVID Bioinformatics Resources 6.8 [39]. Pathway enrichment analysis was performed on the Kyoto Encyclopedia of Genes and Genomes (KEGG; http://www.Genome.Jp/keg), meaningful enrichment of biological process (BP), molecular function (MF), and cellular component (CC), and KEGG pathway was selected with a cut-off of false discovery rate (FDR) <0.05.

### PPI network analysis

PPI network analysis of all the DEGs in cell cycle pathway on the STRING online database (http://string-db.org) [13]. The PPI correlation coefficient was imported into Cytoscape 3.5.1 for visualization [40]. Each node in Cytoscape represents a gene, the edges width between the nodes represent the interaction between them, and the degree represents the number of edges.

### Overall survival analysis based on MM patient

GSE24080 dataset was selected as the validation dataset, which includes clinical and overall survival information of 313 (IgG) patients. The details of this dataset as below: a. total number: 313 newly diagnosed MM patients; b. gender (male/female): 195/118; c. median age: 57.75 (29.70~76.50). The expression level of each hub-gene was divided into high expression group and low expression group according to the mean value. Finally, GraphPad Prism 8 (version 8.2.1) was used to make the Kaplan-Meier Survival Curve. Log-Rank test was used to calculate the OS of above MM patients. The difference was statistically significant at P <0.05.

### Hub-genes expression validation and ROC analysis

Due to GSE24080 dataset lack of NPC and MGUS patient specimen, GSE47552 dataset, 5 Normal Plasma Cells (NPC) as health control, 20 MGUS patients, and 41 MM patients, all patient samples are newly diagnosed without treatment, was used for the hub-genes expression validation and ROC analysis. The area under the curve (AUC) is a key indicator to reflect the accuracy and specificity of the diagnoses.

### Validation of CHK1 expression by real-time quantitative reverse transcription polymerase chain reactions (qRT-PCR)

To measure the gene expression of CHK1, we detected the expression of CHK1 MM bone marrow mononuclear cells (BMNCs) samples using qRT-PCR, including 13 HC, 15 newly diagnosed MM, and 15 R/R MM samples. Total RNA was extracted from BMSCs samples of HC and MM using TRIzol reagent (Invitrogen, Germany) and stored at −80 °C until use. RNA purity and concentration were determined by Thermo Scientific Multiskan GO. RNA samples were reversely transcribed into cDNA using a Primescript RT master mix with Oligo dT primers and random primers in accordance with manufacturer's protocols (CWBIO). Then, the qRT-PCR was performed by SYBR Premix Ex Taq™ II (CWBIO) and StepOne Software v2.1 according to manufacturer's instructions. Primers of detected CHK1 and GAPDH were designed and synthesized by Tsingke (Shanghai, China). And the primer sequences were as shown in Table 3. 2-^ΔΔCt^ value was used to reflect the expression level of CHK1.

**Table 3:** The primer sequences of CHK1 and GAPDH.

**Name**	**Forward primer (5′-3′)**	**Reverse primer (5′-3′)**
CHK1	ACCAGATGCTCAGAGATTCTTCCA	TGAGGTTATCCCTTTCATCCAACAG
GAPDH	ACAACTTTGGTATCGTGGAAGG	GCCATCACGCCACAGTTTC

### Western blotting analysis

To validate the protein expression of CHK1, immunoblot analysis was adopted to evaluate the differential expression of CHK1 in 3 ND MM and 3 R/R MM and every single sample repeat three times. BMNCs samples obtained from ND MM and R/R MM patients and immediately frozen in liquid nitrogen and stored at -80 °C. Then The samples were centrifuged at 12,000 rpm at 4 °C for 30 min. The supernatants were collected and protein amounts were quantified by BCA method. Lysates containing 20 μg of protein was boiled at 95 °C in SDS sample buffer for 10 min, electrophoresed on 12% SDS PAGE gels, and transferred to polyvinyldifluoridine membranes. Subsequently membranes blocked in 5% (w/v) skimmed milk solution for 2h at room temperature and incubated overnight at 4 °C with primary antibody. Anti-CHK1 mouse monoclonal antibody (diluted 1:1000, Cell Signaling Technology, USA) and anti-GAPDH rabbit monoclonal antibody (diluted 1:1000, Beyotime Biotechnology, China) were used. After three washings for 30 min in TBST buffer, membranes were incubated at room temperature for 1h, horseradish peroxidase-conjugated goat anti-mouse or anti-rabbit IgG (diluted 1:1000; Beyotime Biotechnology, China) was used as a secondary antibody and washed three times for 30 min with TBST buffer. Then membranes were developed with ECL reagents and the chemiluminescence signal was imaged using a MiniChemiTM (Sagecreation, China). Immunoblots were quantified using quantity-one software (Bio-Rad, USA).

## Supplementary Material

Supplementary Tables
