# Supplementary material for: CD138- multiple myeloma cells express high level of CHK1 which correlated to overall survival in MM patient
Source: Aging (Albany NY). 2020 Nov 10;12(22):23067–81. doi: 10.18632/aging.104066 (PMC7746343; doi:10.18632/aging.104066)
Supplement: Supplementary Tables [file aging-12-104066-s001..pdf]

SUPPLEMENTARY TABLES

Supplementary Table 1. DEGs analysis result.

NO.	genesymbol	ID	logFC	AveExpr	t	P.Value	adj.P.Val	B
2660	CDC7	7902913	1.20992275	5.37396213	2.78602574	0.02154565	0.1726192	-3.2848305
2701	CDK1	7927710	1.1229915	6.12909975	2.94803956	0.01658302	0.16063097	-3.0362356
2965	CHEK1	7945014	1.25384825	8.16880263	4.37013511	0.0018745	0.09430518	-0.9544463

Supplementary Table 2. Kegg pathway.

Term	Count	PValue	Genes	Fold Enrichment	Bonferroni	Benjamini	FDR
hsa04110:Cell cycle	27	1.01E-06	YWHAZ, E2F5, ANAPC13, PRKDC, ANAPC10, CHEK1, CDC16, CCNE2, RAD21, STAG2, CDC7, CDC6, CDK1, RBL2, ANAPC4, RBL1, YWHAB, SMAD4, CDC23, CDK6, SMAD2, RB1, ATM, SMC3, MCM6, HDAC2, MAD2L1	2.898047493	1.71E-04	1.71E-04	0.001229924
hsa03050:Proteasome	13	1.15E-04	PSMA2, PSMA1, PSMC6, PSMD14, PSMB1, PSMD12, PSMA5, PSMA4, PSMC2, PSMA3, PSMD1, POMP, PSMD6	3.711053725	0.019301519	0.009697783	0.139771344
hsa04114:Oocyte meiosis	21	1.38E-04	CDK1, YWHAZ, ANAPC13, ANAPC4, YWHAB, PPP3R1, CDC23, ANAPC10, CDC16, PPP1CC, PPP1CB, SMC3, CCNE2, MAPK1, MAD2L1, SLK, PPP2CA, PPP2CB, PPP3CB, FBXO5, PPP3CA	2.561405613	0.023147852	0.007776264	0.167929275
hsa04120:Ubiquitin mediated proteolysis	24	1.57E-04	XIAP, UBE3A, ANAPC13, VHL, UBE2G1, ANAPC4, CDC23, UBA6, HERC4, ANAPC10, CDC16, UBE2H, BIRC2, CUL3, CUL2, CUL5, FBXW7, UBE2K, UBA2, UBA3, UBE2W, RCHY1, UBE2D1, TRIP12	2.350403482	0.026260353	0.006630747	0.190790462
hsa03018:RNA degradation	14	2.07E-04	DIS3, PAPOLA, EXOSC8, CNOT8, CNOT6L, LSM5, CNOT2, LSM3, CNOT7, MPHOSPH6, LSM1, HSPA9, C1D, DDX6	3.295375642	0.034576002	0.007012874	0.252202896
hsa03022:Basal transcription factors	10	7.81E-04	TAF11, TAF2, TAF13, GTF2A1, GTF2A2, TAF9B, GTF2H3, GTF2B, TBPL1, GTF2H1	3.833396155	0.124452204	0.021907392	0.949245571
hsa00563:Glycosylphosphatidylinositol(GPI)-anchor biosynthesis	7	0.00853127	PIGK, PIGF, PIGY, PIGX, PIGW, PIGB, PIGN	3.756728232	0.766958304	0.187855372	9.92491482
hsa00520:Amino sugar and nucleotide sugar metabolism	9	0.014340569	PGM2, PGM3, GNPDA2, GFPT1, GNPNAT1, HEXB, UGDH, FPGT, UGP2	2.744363157	0.914183677	0.264307654	16.15657625
hsa00510:N-Glycan biosynthesis	9	0.018550464	STT3B, MAN1A2, ALG10B, DPM1, ALG5, ALG6, MAN1A1, ALG11, ALG8	2.625043019	0.958547035	0.297906315	20.42243587
hsa04914:Progesterone-mediated oocyte maturation	13	0.024178803	CDK1, GNAI3, PIK3CB, ANAPC13, ANAPC4, CDC23, ANAPC10, CDC16, PPP1CB, MAPK1, MAD2L1, MAPK14, MAPK9	2.028134012	0.984406552	0.340379394	25.81453929

hsa00620:Pyruvate metabolism	8	0.026081166	ME1, LDHB, ME2, PDHA1, DLAT, ACAT1, ALDH9A1, MDH1	2.683377309	0.988808972	0.335301738	27.55979969
hsa04210:Apoptosis	13	0.026240681	CFLAR, XIAP, PIK3CB, PPP3R1, BIRC2, ATM, IRAK4, CASP6, CASP3, PRKAR1A, PPP3CB, PPP3CA, CHUK	2.004822127	0.989116299	0.313883203	27.70441471
hsa05120:Epithelial cell signaling in Helicobacter pylori infection	11	0.027367152	ATP6V1C1, ATP6V1A, CASP3, ADAM10, LYN, MAPK14, MAP2K4, MAPK9, ATP6V1B2, ATP6V1D, CHUK	2.170378706	0.991060391	0.304320642	28.71814593
hsa00970:Aminoacyl-tRNA biosynthesis	8	0.029517008	TARS, NARS, DARS, RARS, NARS2, GARS, SEPSECS, FARSB	2.617929082	0.993863117	0.304981642	30.61670827
hsa00270:Cysteine and methionine metabolism	7	0.036878177	LDHB, CTH, MTAP, AHCYL1, MAT2B, ENOPH1, AMD1	2.762300171	0.99831807	0.346788431	36.77138752
hsa05014:Amyotrophic lateral sclerosis (ALS)	9	0.040165789	CASP3, DERL1, MAPK14, RAB5A, PPP3CB, PPP3R1, PPP3CA, CAT, SOD1	2.278339224	0.999059501	0.353103365	39.35473238
hsa00280:Valine, leucine and isoleucine degradation	8	0.041615194	ACADSB, ACADM, OXCT1, ACAT1, HIBADH, PCCA, ALDH9A1, HADHB	2.439433917	0.999272578	0.346268819	40.46256723
hsa04140:Regulation of autophagy	7	0.041831221	GABARAPL2, ATG4C, ATG5, BECN1, ATG4A, PRKAA1, ATG3	2.683377309	0.999299928	0.332071236	40.62608511
hsa04130:SNARE interactions in vesicular transport	7	0.059109694	VAMP7, STX16, BET1, VTI1B, VAMP3, GOSR1, SNAP23	2.471531732	0.999968258	0.420246165	52.44675687
hsa04720:Long-term potentiation	10	0.062803114	MAPK1, NRAS, PPP3CB, PPP3R1, PPP1R12A, RAP1A, RAP1B, PPP3CA, PPP1CC, PPP1CB	1.973071551	0.999983735	0.423814781	54.67467678
hsa00071:Fatty acid metabolism	7	0.072677468	ACADSB, ACADM, ACSL4, ACSL3, ACAT1, ALDH9A1, HADHB	2.347955145	0.999997313	0.457092772	60.16898459
hsa00640:Propanoate metabolism	6	0.084727655	LDHB, ACADM, SUCLG2, ACAT1, PCCA, ALDH9A1	2.515666227	0.999999709	0.49546768	66.0436841
hsa00900:Terpenoid backbone biosynthesis	4	0.095124864	HMGCR, IDI1, ACAT1, PDSS2	3.577836412	0.999999958	0.522323875	70.46147324
